# *In situ* Fabrication of *α*-Bi_2_O_3_/(BiO)_2_CO_3_ Nanoplate Heterojunctions with Tunable Optical Property and Photocatalytic Activity

**DOI:** 10.1038/srep23435

**Published:** 2016-03-21

**Authors:** Yu Huang, Wei Wang, Qian Zhang, Jun-ji Cao, Ru-jin Huang, Wingkei Ho, Shun Cheng Lee

**Affiliations:** 1 Key Laboratory of Aerosol Chemistry and Physics, Institute of Earth Environment, Chinese Academy of Sciences, Xi’an 710061, China; 2 State Key Lab of Loess and Quaternary Geology (SKLLQG), Institute of Earth Environment, Chinese Academy of Sciences, Xi’an 710061, China; 3 Department of Science and Environmental Studies, The Hong Kong Institute of Education, Hong Kong, China; 4 Department of Civil and Environmental Engineering, The Hong Kong Polytechnic University, Hung Hom, Hong Kong

## Abstract

Exploring the full potential use of heterojunction photocatalysts containing bismuth has attracted considerable interest in recent years. Fabrication of well-defined heterojunction photocatalysts with precise modulation of their chemical composition is crucial for tuning their optical properties and photocatalytic activity. In this study, we fabricated nanoplate *α*-Bi_2_O_3_/(BiO)_2_CO_3_ heterojunctions through *in situ* thermal treatment of (BiO)_2_CO_3_ nanoplates synthesized using a facile hydrothermal process. Characterization results showed that the as-prepared Bi_2_O_3_/(BiO)_2_CO_3_ heterojunctions possessed distinct crystal interface and exhibited pronounced structural and optical modulation, resulting in significant improvement of their photocatalytic activity for NO removal under simulated solar light irradiation compared with pristine (BiO)_2_CO_3_. Electron spin resonance spectroscopy showed that ⋅OH radicals were the major reactive species involved in NO degradation, which is consistent with the theoretical analysis. The heterojunction formation can not only broaden the light absorption range but also improve the charge separation of photo-induced electron–hole pairs. This study is an important advancement in the development of semiconductor heterojunctions towards achieving functional photocatalysts.

Nitrogen oxides (NO_x_) are one of the most important precursors for secondary organic aerosol formation that have crucial contributions to the haze events in China^1^. Therefore, NO_x_ abatement is one of the priority issues that need to be addressed for efficient air pollution control. Photocatalysis has emerged as a promising technology for degradation of various pollutants since the pioneer work of Frank *et al*. in 1976^2^. This process involves utilization of infinite solar energy by semiconductors to produce excited electron–hole pairs, which initiate the production of active free radicals and subsequent decomposition of recalcitrant substances under mild conditions. However, the efficiency is greatly restricted by the low light absorption capacity and fast electron–hole recombination of photocatalysts during the reaction processes^3^.

Fabrication of heterojunction photocatalysts with proper band alignment is considered a promising approach to settle these drawbacks *via* band structure reconstruction^4^. Construction of heterogeneous photocatalysts with multiple integrated functional components at different energy levels can regulate the light absorption threshold of materials. However, heterogeneous photocatalysts can also promote quick charge-carrier transport driven by the inner built electric filed at the hetero-interface, which eventually enhances the photocatalytic efficiency^5^,^6^. Bismuth-layered compounds have recently received remarkable attention owing to their unique thin-layered structure, which benefits photo-induced charge separation and thus improves quantum yields^7^. A series of bismuth-based heterojunction photocatalysts, including BiVO_4_/Bi_2_O_3_^8^, Bi_2_O_3_/Bi_2_O_4−x_^9^, and Bi_2_S_3_/BiOI^10^ has been reported and applied for organic pollutant degradation^8^,^9^,^11^,^12^,^13^,^14^. Bismuth subcarbonate ((BiO)_2_CO_3_)^15^ is a non-toxic and environmentally friendly photocatalyst composed alternate Bi_2_O_2_^2+^ and CO_3_^2−^ layers; this material exhibits comparable photocatalytic activity with commercial P25 photocatalyst and has attracted much more attention in recent years. However, (BiO)_2_CO_3_ can only be excited by ultraviolet light, thereby restricting its efficiency for solar energy conversion. Therefore, various heterojunction photocatalysts such as Bi_2_S_3_/(BiO)_2_CO_3_^11^, (BiO)_2_CO_3_/BiOI^12^, and Bi/(BiO)_2_CO_3_^13^ have been constructed to extend the optical absorption and improve the quantum yield of (BiO)_2_CO_3_. However, the synthesis strategies used to fabricate heterojunction structures normally involve two main steps. First, the pristine component is prepared and then deposited with another component to construct heterojunctions. Uniform structures with well-defined morphologies are difficult to achieve because of compatibility issues among different components. Therefore, constructing heterojunction structures with homogeneous distribution of components remains challenging^11^,^12^,^13^.

In this study, a facile calcination approach was used to prepare composition-controllable *α*-Bi_2_O_3_/(BiO)_2_CO_3_ nanoplates with tunable optical properties and photocatalytic activities for the first time. The photocatalytic performance of the heterojunction was evaluated for gaseous NO removal under simulated solar light irradiation. The synthesized *α*-Bi_2_O_3_/(BiO)_2_CO_3_ showed extended light absorption, improved charge separation, and superior photocatalytic activity under simulated solar light irradiation.

## Results

**Figure 1:**
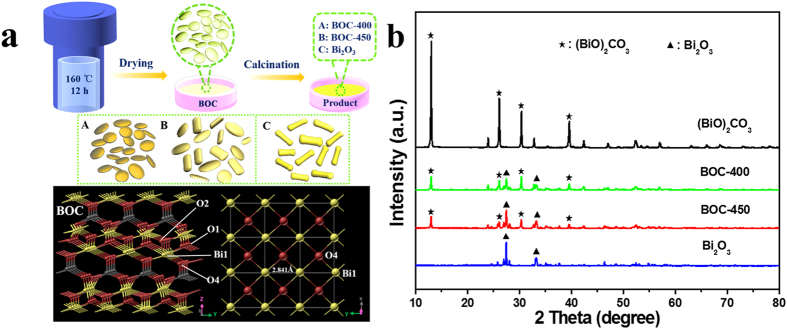
(**a**) Schematic of the fabrication of BOC-based heterojunctions. (**b**) XRD patterns of the as-prepared samples.

As described in Fig. 1a, the *α*-Bi_2_O_3_/(BiO)_2_CO_3_ heterojunction photocatalysts were prepared using a simple hydrothermal procedure, followed by calcination treatment. The thermal stability and calcination temperature of the (BiO)_2_CO_3_ precursor were investigated using thermogravimetric analysis (TGA), and the results showed that (BiO)_2_CO_3_ thermally decomposes to Bi_2_O_3_ at an elevated temperature (>350 °C) through one stage with two different decomposition rates (Fig. S1). The total weight loss was about 5.7% from 350 °C to 430 °C. Weight loss continuously took place when the temperature reached 430 °C. The phase transformation corresponding to *β*-Bi_2_O_3_ → *α*-Bi_2_O_3_ proceeded, which absorbed heat, and then decelerated decomposition. The total weight loss was about 3.5% and 9.2% from 430 °C to 570 °C and 350 °C to 570 °C, respectively. Taking dehydration into account, the weight loss was in good agreement with the reaction: (BiO)_2_CO_3_ → Bi_2_O_3_ + CO_2_, Δm = −8.6% (referenced to (BiO)_2_CO_3_). Based on the thermal decomposition reaction of (BiO)_2_CO_3_, gaining *α*-Bi_2_O_3_/(BiO)_2_CO_3_ composite phases was expected to be gained through precise control of the calcination time and temperature. The crystalline phase information of each sample was determined using X-ray diffraction (XRD) analysis (Fig. 1b). The hydrothermally synthesized white precursor can be accurately indexed to a pure tetragonal phase of bismutite ((BiO)_2_CO_3_; JCPDS file No. 41-1488). The heat treatment of (BiO)_2_CO_3_ at 500 °C for 4 h led to the formation of yellow *α*-Bi_2_O_3_. This formation is demonstrated in the corresponding XRD pattern, in which the dominant peaks of monoclinic *α*-Bi_2_O_3_ are present (JCPDS file No. 71-2274). *α*-Bi_2_O_3_ belongs to the space group P21/*c*, with lattice constants of a = 5.850 Å, b = 8.170 Å, and c = 7.512 Å. Calcination of the (BiO)_2_CO_3_ precursor at 400 °C yielded the *α*-Bi_2_O_3_/(BiO)_2_CO_3_ composite, as evidenced by the typical peaks corresponding to each material in the pattern. The *α*-Bi_2_O_3_/(BiO)_2_CO_3_ composite was maintained with the slight change of peaks intensity as the calcination temperature further increased to 450 °C. This finding indicates the variation in the contents of these two components. Bi_2_O_3_ was designated as *α*-Bi_2_O_3_ when no special instructions were required, and the *α*-Bi_2_O_3_/(BiO)_2_CO_3_ composite was named as BOC-X composite thereafter (X: calcination temperature). Based on the elemental analysis result, the (BiO)_2_CO_3_ contents on the surface of BOC-400 and BOC-450 samples were 54.99% and 43.60%, respectively. The mass contents of the (BiO)_2_CO_3_ phases of Bi_2_O_3_ calcination were estimated to be 5.30% (Table S1).

**Figure 2:**
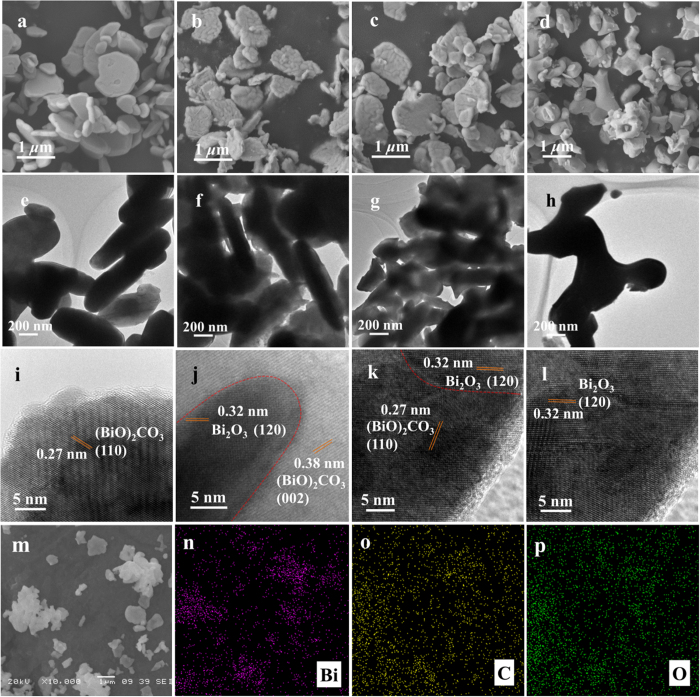
Morphological characterization of the as-prepared samples. (**a–d**) SEM, (**e–h**) low-magnification, and (**i–l**) HR-TEM images of (BiO)_2_CO_3_, BOC-400, BOC-450, and Bi_2_O_3_, respectively. (**m**) SEM image of BOC-450 heterojunction and the corresponding (**n–p**) elemental X-ray mapping for bismuth, carbon, and oxygen elements.

The morphology of the as-prepared samples was characterized through scanning electron microscopy (SEM) and transmission electron microscopy (TEM). Figure 2 shows the two-dimensional plate-like feature of (BiO)_2_CO_3_. The plate-like morphology was not significantly affected after calcination at 400 °C for 1 h. However, the material surface became rougher, and a broad bean-like texture appeared (Fig. 2a,b). As the calcination temperature further increased to 500 °C, the plate-like structure was completely destroyed and transferred to irregular peanut-like shape of Bi_2_O_3_ (Fig. 2d). As reported by Yu *et al*. the changes of the surface microstructures of Ag_2_O/Ag_2_CO_3_ catalyst were observed compared with pristine Ag_2_CO_3_, which was attributed to the phase transformation from Ag_2_CO_3_ to Ag_2_O^15^. The BOC-based results are similar to this analysis. Therefore, it was believed that the change of the shape of BOC-400 and BOC-450 can be attributed to the phase transformation from (BiO)_2_CO_3_ to Bi_2_O_3_. TEM images confirmed that (BiO)_2_CO_3_ had a nanoplate structure with larger dimensions, whereas high-temperature calcination resulted in shrinking of the particle size. As characterized by SEM and TEM, the dimension of pristine (BiO)_2_CO_3_ is about 0.3–1 μm and about 50 nm in thickness. The plate-like morphology was not significantly affected for the sample BOC-400 and BOC-450 which were obtained by the calcination of (BiO)_2_CO_3_ at 400 °C and 450 °C for 1 h, respectively. High-resolution TEM (HR-TEM) images of the as-prepared samples were recorded and shown in Fig. 2i–l. Consistent with the XRD results, distinct lattice fringes were observed in (BiO)_2_CO_3_ and Bi_2_O_3_. For BOC-based composites, the lattice fringes corresponding to the (120) plane of Bi_2_O_3_ were resolved, and the distinct interface between the two components were observed, indicating successful formation of the Bi_2_O_3_ phase over (BiO)_2_CO_3_. X-ray mappings of BOC-450 illustrated in Fig. 2n–p further confirmed that the distribution of C element on the BOC-450 sample (Fig. 2m) is consistent with Bi and O elements on the BOC-450 sample, suggesting that Bi, C, and O are uniformly distributed in the BOC-based heterojunction obtained through *in situ* fabrication.

The Brunauer–Emmett–Teller (BET) surface areas of (BiO)_2_CO_3_, BOC-400, BOC-450, Bi_2_O_3_, and mechanical mixture containing the same components with BOC-400 were determined to be 1.93, 4.32, 0.96, 0.66, and 1.25 m^2^/g, respectively (Table S2). The evident increase in the surface area of BOC-400 was associated with morphological changes and decreased particle sizes. The partial destruction of the hierarchical microspheres resulted in decreased surface area (Fig. 2c,d).

**Figure 3:**
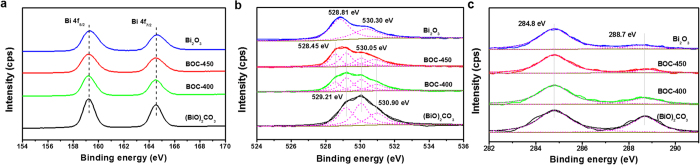
High-resolution XPS spectra of (BiO)_2_CO_3,_ BOC-400, BOC-450, and *α*-Bi_2_O_3_ samples. (**a**) Bi 4f, (**b**) O 1s, and (**c**) C 1s.

The surface chemical states and composition of the as-prepared (BiO)_2_CO_3_, Bi_2_O_3_, and BOC-based composites were characterized using survey and high-resolution X-ray photoelectron spectroscopy (XPS). The survey spectra of the as-prepared samples showed the presence of bismuth, carbon, and oxygen elements (Figure S2). In the high-resolution XPS spectra of Bi 4f orbitals, all samples showed the same binding energy at 164.5 and 159.2 eV, which correspond to Bi 4f_5/2_ and Bi 4f_7/2_, respectively. This finding demonstrates that the main chemical state of bismuth in all samples is +3 (Fig. 3a)^16^,^17^. Figure 3b shows the O 1s spectrum in which peaks at 529.21 and 530.05 eV are assigned to the Bi−O bonds in (BiO)_2_^2+^ and C−O bonds in CO_3_^2−^ of (BiO)_2_CO_3_, respectively^18^,^19^. The peak at 528.81 eV is assigned to Bi−O bond in Bi_2_O_3_. Peaks located at 530.90 and 530.30 eV are assigned to the adsorbed H_2_O on the surface^20^. The O 1s peak in the BOC-based heterojunction contains four shoulders, which can be distinguished in the spectrum and indicate the four chemical states of oxygen present. The peak drift at 528.45 eV of the Bi−O bond in Bi_2_O_3_ can be attributed to the change of inner electron density caused by the formation of BOC-based heterojunction^21^,^22^. Figure 3c shows the C 1s spectrum in which peak at 284.8 eV is attributed to the adventitious carbon species. The peak at 288.7 eV is the characteristic peak of CO_3_^2−^ in (BiO)_2_CO_3_^13^. The intensity of C 1s and O 1s corresponds to the decreasing order of (BiO)_2_CO_3_ → *α*-Bi_2_O_3_/(BiO)_2_CO_3_ → Bi_2_O_3_. The carbon element in Bi_2_O_3_ is mainly ascribed to the adventitious hydrocarbon, and there are also a little amount of (BiO)_2_CO_3_ exist.

**Figure 4:**
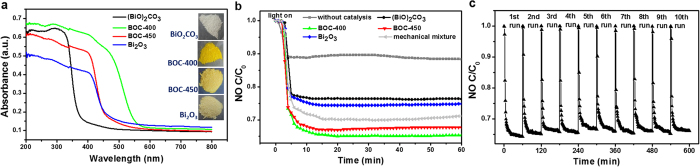
Optical absorption and photocatalytic property of the as-prepared samples. (**a**) UV–visible diffuse reflectance spectra of the as-prepared samples. The insets show photos of the sample color. (**b**) Photocatalytic activities of (BiO)_2_CO_3_, BOC-400, BOC-450, and Bi_2_O_3_ and the mechanical mixture composed of 54.99% (BiO)_2_CO_3_ and 45.01% Bi_2_O_3_ under solar light irradiation for NO removal. (**c**) Recycling property of the composite photocatalyst BOC-400 under solar light irradiation.

UV–visible absorption spectra were obtained to determine the optical properties of the as-prepared samples. Figure 4a presents the UV–visible absorption spectra, in which the absorption range of the as-prepared samples significantly varied. The absorption spectrum of (BiO)_2_CO_3_ was mainly located in the ultraviolet region below 400 nm. BOC-400 exhibited the largest red shift toward 570 nm, whereas BOC-450 and *α*-Bi_2_O_3_ showed a slight red shift to around 470 nm. The absorption intensity of the BOC-based composites in the UV region increased with decreasing Bi_2_O_3_ content. This finding implies that (BiO)_2_CO_3_ performs a pivotal function in the optical property of BOC-based heterojunctions. This trend coincides with the color differences shown in the inset image of Fig. 4a. The obtained powders presented a gradual color evolution from white to bright yellow to pale yellow, which indicates their different light-responsive properties. The absorption of the mechanical mixture containing the same components with BOC-400 also was obtained for comparison (Fig. S3a). From Fig. S3a, it can be found that the absorption edge of the mechanical mixture located at ~470 nm, and exhibited distinct optical absorption ability with BOC-400. Assuming that the samples ((BiO)_2_CO_3_ and Bi_2_O_3_) are indirect semiconductors, a plot of (αhν)^1/2^ versus the energy of absorbed light can be used to determine the bandgaps (Fig. S3b). The bandgap energy of (BiO)_2_CO_3_ and Bi_2_O_3_ estimated from the intercept of the tangent to the plot were 3.04 and 2.33 eV, respectively. The optical absorption results demonstrate that the light-response ability can be tuned by the chemical components of BOC-based heterojunction.

The photocatalytic activities of the as-prepared samples were evaluated by the removal of gaseous NO under simulated solar light irradiation. Figure 4b presents the variations in NO removal rates with increasing irradiation time in the presence of artificial solar light over different as-prepared photocatalysts. The NO removal rates were ignorable without the presence of photocatalyst under simulated solar light irradiation. Pristine (BiO)_2_CO_3_ and Bi_2_O_3_ exhibited moderate photocatalytic activities with removal efficiencies of 23.6% and 25.2%, respectively. However, BOC-400 and BOC-450 were capable of removing 34.9% and 32.6% of NO within 10 min, respectively. This finding suggests that photocatalytic activity was considerably enhanced by the fabricated heterostructure. For comparison, mechanically mixed samples containing 54.99% (BiO)_2_CO_3_ and 45.01% Bi_2_O_3_, which are similar with BOC-400 components, were prepared by physically mixing the two pure components together. The photocatalytic activities of the mechanically mixed samples were much lower than those of BOC-based heterojunction under other identical conditions, indicating that the heterojunction interface plays an important role on the photocatalytic property. As shown in Fig. S4, NO removal under visible light showed a similar trend with that under solar light in the same system. The activity of the samples slightly declined with increasing irradiation time, which can be attributed to the generation of specific intermediates (HNO_2_ and HNO_3_) during the photocatalytic process on the catalyst surface^14^,^23^.Ten cycles of repeated experiments with the used BOC-400 were carried out to further test the stability of the BOC-based heterojunction on photocatalytic NO removal. Multiple runs of photocatalytic experiments showed that BOC-400 was not significantly deactivated during long-term NO oxidation (Fig. 4c), indicating that the BOC-based heterojunction is a stable photocatalyst with promising application in gaseous NO_x_ abatement.

The amount of HNO_2_ or HNO_3_ released into humid air during photocatalysis under solar light was measured using ion chromatography (IC), and the results are shown in Table S2. The NO_3_^−^ of (BiO)_2_CO_3_, BOC-400, BOC-450, and Bi_2_O_3_ were 81.9841, 230.9105, 136.0915, and 100.4016 *μ*g/g, respectively (Table S2). The trend in the total NO_2_^−^ and NO_3_^−^ amounts released from different samples, as determined using IC analysis, was in accordance with their photocatalytic activity. Furthermore, the amounts of NO_2_^−^ were considerably less than that NO_3_^−^, indicating that BOC-based is a high-quality photocatalyst.

## Discussion

**Figure 5:**
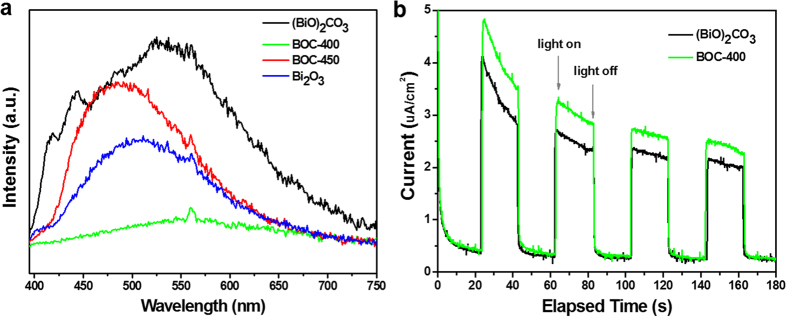
(**a**) Room-temperature PL spectra of the as-prepared samples. (**b**) Photocurrent plot of (BiO)_2_CO_3_ and BOC-400 photoeletrodes.

The formed BOC-based heterojunctions exhibited enhanced photocatalytic activities not only under solar light irradiation but also under visible light irradiation. Photocatalytic reaction is generally a complex physical and chemical process and can be simplified into three steps: generation/separation, transfer, and consumption of photo-generated carriers^24^. The photoluminescence (PL) emission of the as-prepared samples was determined to verify the separation capacity of the photo-generated carriers in the heterojunctions (Fig. 5a). A low PL intensity generally indicates high separation efficiency of the photo-generated electron–hole pairs^25^,^26^. Figure 5a shows that pristine (BiO)_2_CO_3_ and Bi_2_O_3_ exhibited a broad emission peak centered around 425 to 600 nm, which was possibly derived from the direct photo-generated electron–hole recombination of band transition. The as-prepared heterojunctions showed significantly diminished PL intensity compared with the pristine (BiO)_2_CO_3_. The characteristic emission peak around 425 to 600 nm nearly disappeared in the BOC-400 heterostructure; as such, the BOC-based heterojunction presented a remarkable decline in the recombination rate of the photo-generated electron–hole pairs. The photo-responses of (BiO)_2_CO_3_ and BOC-400 electrodes in on-off cycles under solar light irradiation were investigated and the results are shown in Fig. 5b. From Fig. 5b, it can be seen that the photocurrent was drastically increased when the irradiation was turned on, whereas the photocurrent value rapidly decreased as soon as the irradiation was turned off. The photocurrent density generated by BOC-400 electrode (about 3.3 μA/cm^2^) was about 1.3 times of that induced by (BiO)_2_CO_3_ (2.5 μA/cm^2^) under solar light irradiation. This enhanced photocurrent indicated that the calcination treatment of (BiO)_2_CO_3_ precursor could efficiently improve the separation of photo-generated electron-hole pairs. The unique thin-layered structure of (BiO)_2_CO_3_ also benefited from the photo-induced charge separation and transfer of BOC-based heterojunction (Fig. 1a). Efficient charge separation can prolong the lifetime of photo-generated charge carriers, improve the efficiency of interfacial charge transfer to adsorbed substrates, consequently improving the photocatalytic activity^27^.

**Figure 6:**
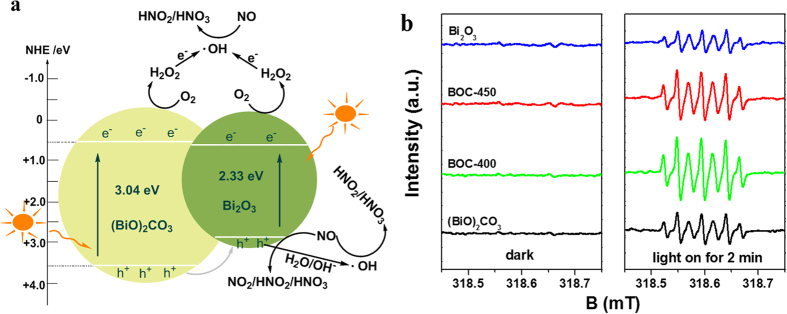
(**a**) Schematic of the energy bands of (BiO)_2_CO_3_ and Bi_2_O_3_ and the transfer of photo-generated charges in BOC-based heterojunctions under simulated solar light (λ > 290 nm) irradiation. (**b**) DMPO-ESR spin-trapping spectra of (BiO)_2_CO_3_, BOC-400, BOC-450, and Bi_2_O_3_ for the detection of hydroxyl radicals (⋅OH) in aqueous solution under UV light irradiation.

The electronic band structure of the obtained samples was investigated through electronegativity calculation to confirm the oxidation capacity of the prepared BOC-based heterojunctions during photocatalytic reactions. The valence band (VB) and conduction band (CB) potentials of (BiO)_2_CO_3_ and Bi_2_O_3_ can be predicted using the following equation:


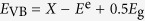



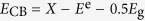


where *X* is the absolute electronegativity of the semiconductor and defined as the geometric mean of the absolute electronegativity of constituent atoms (*X* values for (BiO)_2_CO_3_ and Bi_2_O_3_ are 6.54 and 6.23 eV, respectively), *E*^e^ is the energy of free electrons on the hydrogen scale (ca. 4.5 eV), *E*_VB_ is the VB edge potential, *E*_CB_ is the CB edge potential, and *E*_g_ is the band gap energy of the semiconductor obtained from the UV–visible diffuse reflectance absorption. The top of the VB and bottom of the CB of (BiO)_2_CO_3_ and Bi_2_O_3_ were 3.56/0.52 and 2.89/0.56 eV, respectively. The proposed electronic band structure of the BOC-based heterojunction is depicted in Fig. 6a. With the suitable band gap and VB/CB potentials, Bi_2_O_3_ exhibited more efficient light absorption and photocatalytic activity than (BiO)_2_CO_3_ based on the photocatalysis trend. A type I heterojunction is formed when the two semiconductors form contact. In addition, holes migrated from (BiO)_2_CO_3_ to Bi_2_O_3_ because the VB potential level of (BiO)_2_CO_3_ was more positive than that of Bi_2_O_3_; electrons cannot transfer from one conduction band to the other because of their close conduction band potentials. A built-in potential from (BiO)_2_CO_3_ to Bi_2_O_3_ can generate and reach equilibrium in the dark. When the obtained BOC-based composite was irradiated under solar light, the photo-generated electrons and holes migrated and the charge equilibrium was destroyed. The photo-induced carriers in the corresponding layer reacted with the target NO_x_, partially transferred to the BOC-based hetero-structural interface, and recombined with one another. Therefore, heterojunction generation promotes separation and prolongs the lifetime of photo-generated carriers, resulting in increased removal rates of the obtained composites. This finding is in accordance with the PL emission and photocatalytic properties. When the redox potential of O_2_/H_2_O_2_ is 0.695 eV, the photo-excited electrons of (BiO)_2_CO_3_ and Bi_2_O_3_ can reduce O_2_ to H_2_O_2_, instead of reducing O_2_ into ⋅O_2_^−^ because the CB potential of (BiO)_2_CO_3_ and Bi_2_O_3_ is more positive than that of the redox potential of O_2_/⋅O_2_^−^. As such, the formed H_2_O_2_ is further transformed into ⋅OH by capturing an electron. In addition, the valence band potential of Bi_2_O_3_ (2.89 eV) is more positive than the redox potential of OH^–^/⋅OH (1.99 eV). Therefore, the restructured holes at the valence band of Bi_2_O_3_ can oxidize OH^–^ into ⋅OH radicals. The resulting ⋅OH can oxidize target pollutants to the final products.

The DMPO-ESR spin-trapping spectra of the as-prepared samples were determined to confirm the major factors that confer Bi_2_O_3_/(BiO)_2_CO_3_ heterojunctions with higher light activity than the (BiO)_2_CO_3_ precursor and Bi_2_O_3_. The results are consistent with those obtained from electronegativity calculation and shown in Fig. 6b. ⋅OH radicals were detected in the as-prepared samples under UV light irradiation. Figure 6b shows that the signals assigned to DMPOX are generated from the oxidation of DMPO by two ⋅OH radicals^28^. The experimental results showed that the signal of ⋅OH radicals in the BOC-based heterojunction was considerably stronger than that in the pristine (BiO)_2_CO_3_ and Bi_2_O_3_ (Fig. 6b). The intensity of ⋅OH radicals is associated with the photocatalytic activities of the BOC-based heterojunctions. Therefore, the ⋅OH radicals are the predominant reactive oxidation species generated during photocatalysis.

Studies have shown that NO under low concentrations exhibits long-term stability in air. NO reacts with photo-generated reactive radicals to produce HNO_3_ or N_2_ as the final product at room or low temperature, regardless of the presence of photocatalysts and reductants^23^,^29^,^30^,^31^. Therefore, the possible photocatalytic mechanism of BOC-based heterojunctions can be explained as follows (Fig. 6a): under solar light irradiation, (BiO)_2_CO_3_ and Bi_2_O_3_ can be excited and produce photo-generated electron–hole pairs. As the conduction band of (BiO)_2_CO_3_ (+0.52 eV) is slightly more negative than that of Bi_2_O_3_ (+0.56 eV), the photo-generated electrons in the (BiO)_2_CO_3_ layer cannot transfer to the Bi_2_O_3_ surface. However, the valence band of (BiO)_2_CO_3_ (+3.56 eV) is more positive than that of Bi_2_O_3_ (+2.89 eV), suggesting that the photo-induced holes on the (BiO)_2_CO_3_ surface can migrate to the Bi_2_O_3_ surface. This transfer effectively promotes separation and prolongs the lifetime of the photo-excited carriers, resulting in enhanced photocatalytic activity. After the separation of the photo-induced carriers, two kinds of photo-generated charge carriers are transformed into active species (⋅OH), which are responsible for the removal of gaseous NO. The restructured holes at the VB of Bi_2_O_3_ can directly oxidize NO, considering that the redox potentials of NO_2_/NO, HNO_2_/NO, and HNO_3_/NO are 1.03, 0.99, and 0.94 eV, respectively. Overall, the proposed mechanism of photocatalytic removal of gaseous NO involves reactions displayed in Eqs 1, 2, 3, in which NO reacted with reactive species to produce HNO_2_ and HNO_3_^23^.


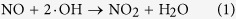



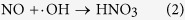



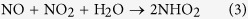


## Conclusion

In summary, *α*-Bi_2_O_3_/(BiO)_2_CO_3_ nanoplate heterojunctions were controllably synthesized by the hydrothermal method combined with *in situ* thermal treatment. The experimental results showed that the photocatalytic performances on NO degradation over *α*-Bi_2_O_3_/(BiO)_2_CO_3_ heterojunctions are remarkably enhanced under solar light irradiation. The characterization results indicated that the enhanced photocatalytic capability is derived from the extended light absorption, promoted separation and prolonged lifetime of the photo-generated carriers, which are attributed to the heterojunction interface formed between pristine (BiO)_2_CO_3_ and Bi_2_O_3_. The photocatalytic mechanism on NO removal was discussed in detail accordingly. In conclusion, fabricating heterojunctions is an important strategy for the development of highly efficient photocatalysts for the removal of gaseous pollutants in air.

## Methods

### Materials

All chemicals used were of analytical grade and used without further purification. Bi(NO_3_)_3_·5H_2_O was purchased from Sigma–Aldrich. Urea (CO(NH_2_)_2_) and ethanol were provided by Sinopharm Chemical Reagent Co., Ltd., China. Distilled water (Milli-Q water) was used throughout the experiments.

### Sample Preparation

Bi_2_O_3_/(BiO)_2_CO_3_ heterojunction photocatalysts were synthesized through the hydrothermal method, followed by calcination under controlled conditions. In a typical procedure, 2.5 mmol of Bi(NO_3_)_3_·5H_2_O and 10 mmol of CO(NH_2_)_2_ were mixed and dissolved into 35 mL of deionized water. After vigorous agitation for 30 min at room temperature, the formed suspension liquid was transferred into a 50 mL Teflon-lined stainless steel autoclave and heated at 160 °C for 12 h. After naturally cooling the autoclave to room temperature, the white (BiO)_2_CO_3_ sample was collected, repeatedly washed with deionized water and absolute ethanol three times, and dried overnight at 70 °C. The as-prepared (BiO)_2_CO_3_ sample was further calcined at 400 and 450 °C for 1 h to obtain Bi_2_O_3_/(BiO)_2_CO_3_ composites (denoted as BOC-400 and BOC-450, respectively). Based on the elemental analysis results, the carbon contents in BOC-400 and BOC-450 were 1.14% and 1.03%, respectively. Therefore, it can be deduced that the mass contents of (BiO)_2_CO_3_ were estimated to be 54.99% and 43.60% in BOC-400, and BOC-450, respectively. A Bi_2_O_3_ sample was also prepared through calcination of the (BiO)_2_CO_3_ precursor at 500 °C for 4 h based on the previous study by Ai *et al*.^32^. The carbon fraction in Bi_2_O_3_ was 0.12%.

### Characterization

Powder XRD patterns were obtained on a PANalytical X’ Pert PRO X-ray diffractometer at 40 kV and 40 mA with Cu K*α* radiation (λ = 1.5406 Å). Elemental analyses were carried out on a Elementar vario EL instrument (German, detection limit: 0.015%, standard deviation: ⩽0.1% abs) with He purging for 20 s before test. TGA was performed using TA instrument SDT Q600 at a heating rate of 10 °C/min from room temperature to 800 °C under air flow (100 mL/min). SEM images were obtained on a JSM-6700 field-emission scanning electron microscope. TEM images were recorded on a JEOL JEM-2010 electron microscope operated at an accelerating voltage of 200 kV. Imaging samples were prepared by ultrasonically dispersing a small amount of the samples in absolute ethanol, and the dispersion was dropped on carbon-coated copper grids. Elemental X-ray mapping was performed using a Si-Li energy-dispersive X-ray detector with an ultrathin window (Thermo Electron Corporation, Noran System SIX, Franklin, MA, USA). The BET surface area was determined using an N-adsorption apparatus ASAP 2020. XPS spectra were recorded on a Thermo ESCALAB 250 with a monochromatic Al K*α* source (Physical Electronics) operated at 150 W. UV–visible DRS spectra were recorded at room temperature using Hitachi U-4100 UV–visible-NIR spectrophotometer equipped with an integrated sphere. PL analysis was conducted on a Hitachi F-7000. DMPO-ESR spin-trapping was performed using a fluorescence spectrometer (FLsp920, Edinburgh Instruments). The sample for ESR measurement was prepared by mixing the as-prepared samples with 50 mM of DMPO (5,5′-dimethyl-1-pyrroline N-oxide) solution to detect free radicals.

### Photocatalytic NO Removal Experiment

Batch experiments were performed in a continuous flow reactor at room temperature to investigate the photocatalytic performance of the prepared photocatalysts for NO removal from air. The rectangular reactor, with a volume of 4.5 L (10 cm × 30 cm × 15 cm, H × L × W), was fabricated from stainless steel and covered with quartz glass. A 300 W commercial Xenon lamp (λ > 290 nm) with and without a 420 nm cutoff filter was used to provide simulated solar light and visible light irradiation, respectively. The sample dish, which was prepared by mixing 0.2 g of the catalyst with deionized water and evaporating at 70 °C to form a uniform coating layer, was placed in the middle of the reactor. The lamp was vertically placed outside the reactor above the sample dish. NO gas was acquired from a compressed gas cylinder at a concentration of 48 ppm NO (N_2_ balance, BOC gas) based on the National Institute of Stands and Technology standard. The initial NO concentration was diluted to around 400 ppb by air stream supplied by a zero-air generator (Sabio Model 1001), and the flow rate was controlled at 3 L/min. The lamp was turned on after adsorption–desorption equilibrium was achieved. The NO concentration was continuously recorded using a chemiluminescence NO_x_ analyzer (EC 9841 series NO_x_) at a sampling rate of 0.6 L/min. The NO removal rate (%) was evaluated with the ratio of NO concentration in the feeding stream relative to that in the outlet stream: *η* (%) = C/C_0_. The NO reaction with air was considered negligible when performing a control experiment with or without light in the absence of a photocatalyst. After the reaction was completed, the intermediate and final products (nitrate and nitrite ions) remaining on the catalyst powders were extracted by immersing the powders into deionized water (6 mL) and measured with an IC Dionex LC20. The mobile phase was composed of a mixture of 1.8 mM Na_2_CO_3_ and 1.7 mM NaHCO_3_ at a flow rate of 1.20 mL/min, and the injected sample volume was 20 *μ*L. Photocatalytic stability tests were performed under identical conditions as the removal efficiency test of gaseous NO. The lamp was turned on after adsorption–desorption equilibrium was achieved. After one hour photocatalytic performance test, the lamp was turned off. After it, the lamp was turned on again once the adsorption–desorption equilibrium was achieved. Carry out the loop for 10 times, the stability test finish.

### Photoelectrochemical measurements

The photoelectrochemical properties of (BiO)_2_CO_3_ and BOC-400 were evaluated using a Parstat4000 electrochemical workstation (Princeton, USA) in a conventional three-electrode cell, in which a platinum plate and Ag/AgCl electrode were used as counter electrode and reference electrode, respectively. In order to fabricate the working electrode, 20 mg (BiO)_2_CO_3_ or BOC-400 was dispersed into 5 mL 1 wt% Nafion ethanol solution to obtain homogeneous suspension through bath sonication. Then (BiO)_2_CO_3_ or BOC-400 films were modified on the fluorine doped tin oxide (FTO) conducting glass by dip coating and dried at room temperature. The current-time curves were measured at 1.2 V vs. Ag/AgCl in 0.1 mol L^−1^ Na_2_SO_4_ at ambient temperature under a 300 W Xe arc lamp.

## Additional Information

**How to cite this article**: Huang, Y. *et al. In situ* Fabrication of *α*-Bi_2_O_3_/(BiO)_2_CO_3_ Nanoplate Heterojunctions with Tunable Optical Property and Photocatalytic Activity. *Sci. Rep.* **6**, 23435; doi: 10.1038/srep23435 (2016).

## Supplementary Material

Supplementary Information
