# Supplementary material for: In situ Fabrication of α-Bi2O3/(BiO)2CO3 Nanoplate Heterojunctions with Tunable Optical Property and Photocatalytic Activity
Source: Sci Rep. 2016 Mar 21;6:23435. doi: 10.1038/srep23435 (PMC4807223; doi:10.1038/srep23435)
Supplement: Supplementary Information [file srep23435-s1.pdf]

Supporting Information

In situ Fabrication of *α*-Bi_2O_3/$(BiO)_2CO_3$ Nano-Plate Heterojunctions with Tunable Optical Properties and Photocatalytic Activity

Yu Huang[1,2,*], Wei Wang[1], Qian Zhang[1], Jun-ji Cao[1,2], Ru-jin Huang[1,2], Wingkei Ho[3,*], and Shun Cheng Lee[4]

[1.] *Key Laboratory of Aerosol Chemistry and Physics, Institute of Earth Environment, Chinese Academy of Sciences, Xi'an 710061, China.*

[2.] *State Key Lab of Loess and Quaternary Geology (SKLLQG), Institute of Earth Environment, Chinese Academy of Sciences, Xi'an 710061, China.*

[3.] *Department of Science and Environmental Studies, The Hong Kong Institute of Education, Hong Kong, China.*

[4.] *Department of Civil and Environmental Engineering, The Hong Kong Polytechnic University, Hung Hom, Hong Kong.*

Submitted to: Scientific Reports

[*]Corresponding authors:

Prof. Yu Huang, E-mail address: huangyu@ieecas.cn

Dr. Wing-kei Ho, E-mail address: keithho@ied.edu.hk

Supplementary figures

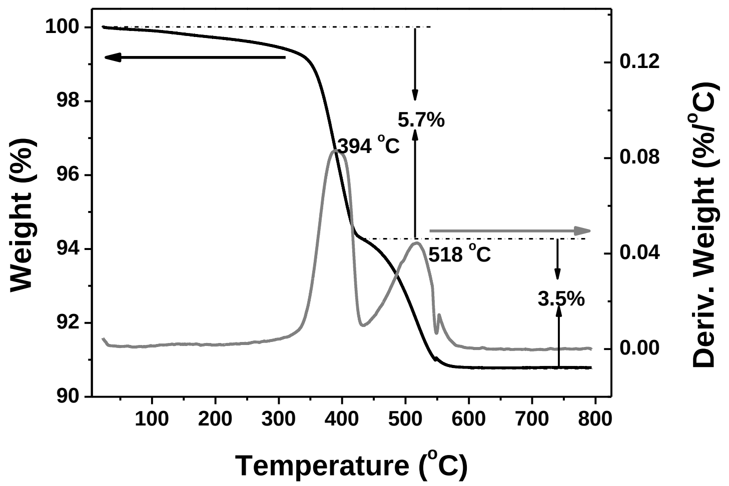

Figure S1 TGA profiles of $(BiO)_2CO_3$ powders in air flow.

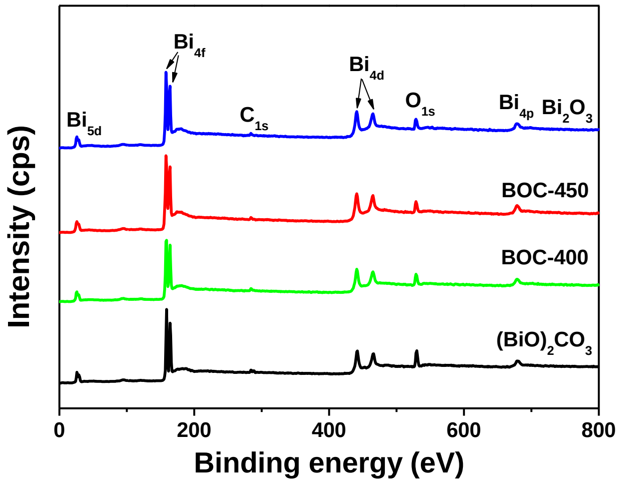

Figure S2 XPS survey spectra of the samples.

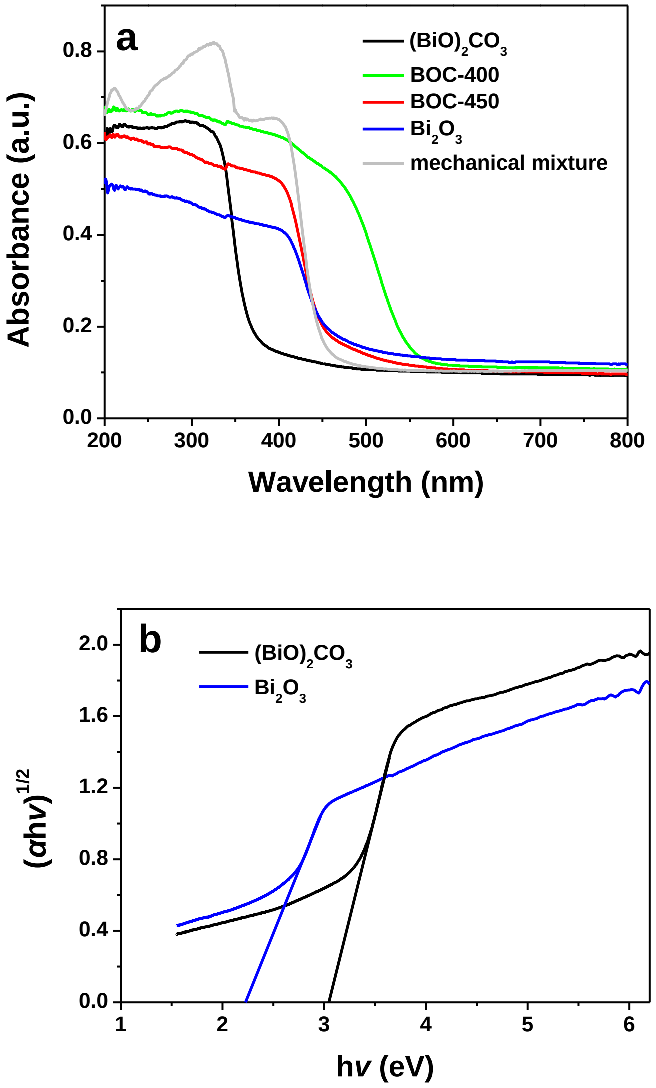

Figure S3 (a) UV–visible diffuse reflectance spectra of the as-prepared samples and the mechanical mixture composed of 54.99% $(BiO)_2CO_3$ and 45.01% Bi_2O_3. (b) Plots of $(\alpha h\nu)^{1/2}$ versus energy ($h\nu$) for the as-prepared samples.

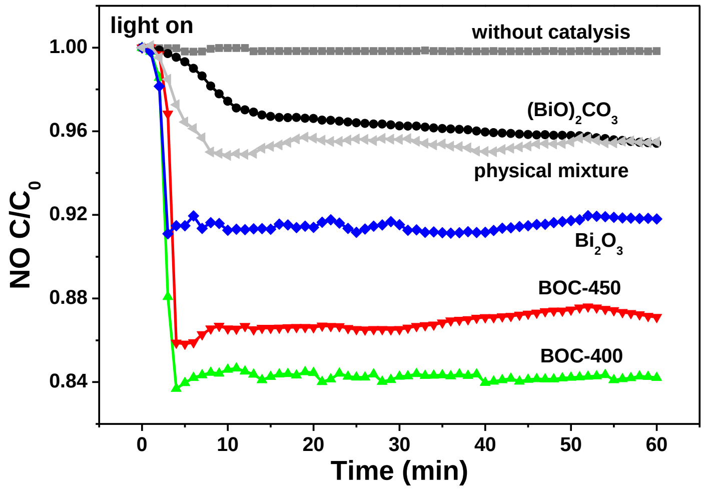

Figure S4 Photocatalytic activities of $(BiO)_2CO_3$, BOC-400, BOC-450, Bi_2O_3 and physical mixture under visible light irradiation for NO removal.

Supplementary Tables

Table S1 Results of Carbon and $(BiO)_2CO_3$ contents in all samples

	$(BiO)_2CO_3$	BOC-400	BOC-450	Bi_2O_3
Elemental analysis C (%) found	2.342	1.137	1.027	0.124
Stoichiometry C (%)	2.353	—	—	0.000
$(BiO)_2CO_3$ (%) calculated from elemental analysis	99.530	54.990	43.600	5.300

Table S2 Results of BET Surface Area and IC

Samples	BET Surface Area (m^2/g)	NO_2^- ($\mu g/g$)	NO_3^- ($\mu g/g$)
$(BiO)_2CO_3$	1.93	5.3558	81.9841
BOC-400	4.32	9.8051	230.9105
BOC-450	0.96	5.4512	136.0915
Bi_2O_3	0.66	18.1312	100.4016
Mechanical mixture	1. 25	—	—
